# Finite-Time Disturbance Observer for Robotic Manipulators

**DOI:** 10.3390/s19081943

**Published:** 2019-04-25

**Authors:** Pengfei Cao, Yahui Gan, Xianzhong Dai

**Affiliations:** 1 School of Automation, Southeast University, Nanjing 210096, China; pengfei_cao@seu.edu.cn (P.C.); xzdai@seu.edu.cn (X.D.); 2 Key Lab of Measurement and Control of Complex Systems of Engineering, Ministry of Education, Nanjing 210096, China

**Keywords:** disturbance observer, robotic manipulator, finite-time observer, human-robot interaction, estimation

## Abstract

Robotic manipulators may be subject to different types of disturbances such as unknown payloads, unmodeled dynamics, and environment interaction forces. Observing these unknown disturbances in robotic manipulators is fundamental in many robotic applications such as disturbance rejection and sensorless force control. In this paper, a novel disturbance observer (DOB) is introduced based on the insights from the finite-time observer (FTO) and robot dynamics. Different from the traditional DOBs, this new observer can provide the capability to track the disturbance within a finite time. The performance of the presented observer is verified by two kinds of typical disturbances for a two-link manipulator with a comparison with several existing DOBs. The simulation results show the rapidity and accuracy of the proposed FTO.

## 1. Introduction

Robotic manipulators could be subject to different kinds of disturbances when they carry on normal operations. Roughly speaking, these disturbances can be divided into two categories, namely external and internal disturbances. These disturbances may present useful information about how the manipulator interacts with its environment. In addition to the interaction force disturbance, adverse disturbances such as unknown payloads, unmodeled dynamics may challenge the performance of the manipulator by affecting the trajectory tracking accuracy. In order to deduce these uncertain disturbances induced by either internal or external factors, it is necessary to incorporate a disturbance observer (DOB) to estimate these disturbances. Once the observer is provided, the DOB in robotic manipulators can enable versatile applications including disturbance observer based control [1,2,3], friction estimation and compensation [4,5], sensorless force/torque control [6,7,8], fault diagnose and isolation (FDI) [9,10,11] etc. For example, the interaction force between human and robot could be treated as the joint torque disturbances as the Cartesian forces could be projected into the joint level. By observing the interaction toque, the manipulator could sense human intention or accidental collision [11,12]. Thus, the design of a well and sound DOB is not only theoretically important to robotic techniques but also empirically required to enhance the performance of robotic manipulators.

The disturbance observer technique has been widely utilized in robotic manipulators for a variety of purposes. The basic idea of DOB is to use the robot motion state and joint torque as input and then to estimate all the unknown internal and external torque imposed on the manipulator in a lumped term as the output. In Reference [5], a nonlinear disturbance observer (NDOB) was established to estimate the rapidly varying friction by elaborately choosing a nonlinear function. However, the selection of such a nonlinear function is nontrivial, and the implementation of this NDOB is limited to a two link condition. By observing the generalized momentum, the generalized momentum observer (GMO) [9] is built, which not only can avoid the calculation of acceleration to decrease the influence of noise in position measurement but also can generate the observation of disturbances as a first-order filtered result of the true value. GMO is able to realize FDI such as the prediction of the accidental impact as well as the saturated actuator fault [9]. Its easy implementation and reliable performance make GMO a popular and widely used method in many robotic applications [6,8,12]. However, in practice, GMO has to make a trade-off between the response rate and noise filtering. In a sensorless scenario, GMO is designed to remove the noise in a motor current as much as possible but at the cost of a delayed response. Such delayed behavior will degrade the detection sensitivity and response rate when GMO is applied in collision detection. As an enhancement of GMO, an external state observer (ESO) was proposed in Reference [10] by taking the disturbance as an external state. Moreover, despite the fact that many DOBs have been developed for robotic manipulators [5,9,10,13,14], the asymptotic convergence rate of these DOBs reveals that the estimation error will not converge to a certain small level quickly and will never decay to zero.

From the aspect of convergence rates, most of the existing DOBs for robotic disturbance achieve the asymptotically tracking performance. Specifically, the system convergence rate of traditional DOBs is at best exponential while FTO can obtain faster convergence rate with a finite-time convergence. Due to the finite-time features, several types of FTOs have been designed for different systems with versatile applications [15,16,17,18,19,20]. In this paper, a novel DOB is conceived by the requirement of a fast and accurate estimation of robotic disturbances. Based on the robot dynamic model, the concept in finite-time control is employed in the observer design. The resulting FTO could enable the estimation of disturbance in a finite time, which guarantees that the estimation error could vanish after a certain time. The proposed FTO also removes the requirement of computing the acceleration. Its finite-time convergent property renders the disturbance observation with more accuracy and quickness.

The rest of the paper is organized as follows. In Section 2, some basic knowledge of robot dynamics and finite-time stability is presented along with the introduction of GMO. Section 3 details the design of the proposed FTO for disturbance estimation. The stability and convergent rate is also presented. Section 4 presents the tracking performance of FTO in a simulation in comparison with other DOBs. The conclusion and future perspective are provided in Section 5.

## 2. Preliminaries

### 2.1. Robot Dynamic Model

For an *n* degrees-of-freedom (DOFs) robotic manipulator with a rigid link and joint, its dynamics could be represented by

(1) $$M\left(q\right)\ddot{q}+C\left(q,\dot{q}\right)\dot{q}+G\left(q\right)+\tau_{f}=\tau +\tau_{d}$$

where $q\in R^{n}$ is the joint position and its first and second derivatives $\dot{q}$ and $\ddot{q}$ are the joint velocity and acceleration, respectively. $M\left(q\right)\in R^{n\times n}$ denotes the symmetric and positive-definite inertia matrix, $C\left(q,\dot{q}\right)\dot{q}\in R^{n}$ represents the centrifugal and Coriolis torque vector with $G\left(q\right)\in R^{n}$ the gravity effect. $\tau_{f}\in R^{n}$ denotes the lumped friction effect from both the motor and link sides and are always described with the following Coulomb-viscous model, namely

(2) $$\tau_{f}=F_{c}\mathrm{sgn}\left(\dot{q}\right)+F_{v}\dot{q}$$

with $F_{c}=\mathrm{diag}\left\{F_{c1},\ldots ,F_{cn}\right\},F_{v}=\mathrm{diag}\left\{F_{v1},\ldots ,F_{vn}\right\}$. $F_{ci},F_{vi}\left(1\leq i\leq n\right)$ are the Coulomb and viscous friction coefficients for the *i*th joint. Such a friction model could capture most dynamic property of the friction in a rigid joint. The equivalent motor torque at the link side through a reduced amplification is denoted as $\tau \in R^{n}$. $\tau_{d}\in R^{n}$ is the internal/external disturbances which could be an external force, unmodeled or uncertain robot dynamics. The exact meaning of $\tau_{d}$ decides on the specific application. The observed disturbance for a manipulator can be further utilized in FDI and disturbance rejection control. For example, $\tau_{d}$ is deemed as the physical impact with the environment for collision detection scenario and, thus, $\tau_{d}$ can indicate the occurrence of the collision. The robot dynamics model in Equation (1) has the following property.

**Property** **1.** *The matrix $\dot{M}\left(q\right)-2C\left(q,\dot{q}\right)$ is skew-symmetry [21], and it follows that*

(3) $$\dot{M}\left(q\right)=C\left(q,\dot{q}\right)+C^{T}\left(q,\dot{q}\right).$$

### 2.2. Disturbance Observer Using Generalized Momentum

In order to estimate the disturbances for a robotic manipulator, different observer design techniques have been envisaged to estimate the disturbances. One of the commonly used observers is the generalized momentum observer (GMO) proposed in Reference [9] of which the basic concept is to observe the generalized momentum $p=M\left(q\right)\dot{q}$. Combined with the generalized momentum *p*, the robot dynamics in Equation (1) could be rewritten to

(4) $$\dot{p}=\tau +C^{T}\left(q,\dot{q}\right)\dot{q}-G\left(q\right)+\tau_{d}.$$

Then, GMO method is implemented as follows:

(5) $$\hat{\dot{p}}=\tau +C^{T}\left(q,\dot{q}\right)\dot{q}-G\left(q\right)+{\hat{\tau }}_{d}$$

(6) $${\hat{\tau }}_{d}=K_{o}\left(p-\hat{p}\right)$$

where $\left(\hat{\cdot }\right)$ denotes the estimated value and $K_{o}=\mathrm{diag}\left\{k_{oi}\right\}>0$. More compactly, the disturbance estimation ${\hat{\tau }}_{d}$ is given as

(7) $${\hat{\tau }}_{d}=K_{o}\left[p-\int (\tau +C^{T}\left(q,\dot{q}\right)\dot{q}-G\left(q\right)+{\hat{\tau }}_{d})dt\right].$$

From Equations (5) and (6), the time evolution of GMO is given by

(8) $${\hat{\dot{\tau }}}_{d}=K_{o}\left(\tau_{d}-{\hat{\tau }}_{d}\right)$$

or equivalently expressed in the Laplace domain as

(9) $${\hat{\tau }}_{d}=\frac{K_{o}}{s+K_{o}}\tau_{d}.$$

Thus, the observation ${\hat{\tau }}_{d}$ can be interpreted as the output of a first-order filtering of $\tau_{d}$. The obtained estimation ${\hat{\tau }}_{d}$ yielded by GMO exponentially converges to the disturbance $\tau_{d}$. It is worth noting that the convergent rate of this observer critically depends on the observance matrix $K_{o}$. In practice, a large value of $k_{oi}$ is demanded to quickly reproduce the disturbance. However, system measurement noises and modeling errors will limit the gain of the observer [10]. Moreover, the estimation error exponentially decays which reveals that the estimation error always exists. Particularly, the estimation error will not decrease to a relatively small level until a certain time elapses, suggesting GMO fails to provide a fast and accurate estimation of the rapidly changing disturbances.

### 2.3. Finite-Time Stability

Consider the following nonlinear autonomous system.

(10) $$\dot{x}=f\left(x\right),x\in R^{n},f\left(0\right)=0$$

where *f* satisfies the locally Lipschitz continuous condition. Some basic knowledge of finite-time stability and homogeneity is recalled hereafter. The definition of finite-time stability (FTS) [22,23] is given as follows.

**Definition** **1.** The equilibrium x=0 of Equation (10) is finite-time convergent if there is a function T:U\{0}→(0,+∞) (U is a neighborhood of the origin) such that every solution trajectory $x(t,x_{0})$ of Equation (10) starting from the initial point $x_{0}\in U\backslash \left\{0\right\}$ is well-defined and unique on $\left[0,T\left(x_{0}\right)\right]$ and $\lim_{t\to T(x_{0})}x\left(t,x_{0}\right)=0$. Here, $T(x_{0})$ is called the settling time of Equation (10). If the equilibrium of Equation (10) is Lyapunov stable and finite-time convergent, then the system in Equation (10) is finite-time stable (FTS).

In addition, the concept of homogeneity [23] is also introduced.

**Definition** **2.** *A function $W:R^{n}\to R$ is homogeneous of degree d with respect to the weights $\left(r_{1},\ldots ,r_{n}\right)\in R^{n}$ with $r_{i}>0\;\left(i=1,\ldots ,n\right)$ if*

(11) $$W\left(\lambda^{r_{1}}x_{1},\ldots ,\lambda^{r_{n}}x_{n}\right)=\lambda^{d}W\left(x_{1},\ldots ,x_{n}\right),\forall \lambda >0.$$

*A vector field f is homogeneous of degree d with respect to the weights $\left(r_{1},\ldots ,r_{n}\right)\in R^{n}$ with $r_{i}>0$(i=1,…,n) if*

(12) $$f_{i}\left(\lambda^{r_{1}}x_{1},\ldots ,\lambda^{r_{n}}x_{n}\right)=\lambda^{r_{i}+d}f_{i}\left(x_{1},\ldots ,x_{n}\right),\forall \lambda >0$$

*where $f_{i}$ is the ith component of f. The system in Equation (10) is said to be homogeneous of degree d if the vector field f is homogeneous of degree d.*

Next, a criteria to determine whether a system is FTS is described in the following theorem.

**Theorem** **1.** [24]: If the origin of Equation (10) is locally asymptotically stable and homogeneous of degree d<0, then it is globally FTS.

## 3. Finite-Time Observer of Robotic Disturbance

The objective of this paper is to design a finite-time observer such that the observation of disturbance $\tau_{d}$ could converge to its actual value within a finite time. In this section, a basic FTO is primarily formulated to estimate the disturbance. Then, taking advantage of the the available system signals, a reduced-order FTO is deduced in order to reduce the computation burden and phase lag. Finally, an improved expression of the FTO is conceived such that the inversion of inertia matrix is avoided.

### 3.1. Finite-Time Observer Design

From Equation (1), the acceleration $\ddot{q}$ can be written as

(13) $$\ddot{q}=M^{-1}\left(q\right)\tau_{d}+M^{-1}\left(q\right)\left(\tau -C\left(q,\dot{q}\right)\dot{q}-G\left(q\right)-\tau_{f}\right).$$

By designating $\tau_{a}=\tau -C\left(q,\dot{q}\right)\dot{q}-G\left(q\right)-\tau_{f}$, the above equation can be rewritten as

(14) $$\ddot{q}=M^{-1}\left(q\right)\tau_{d}+M^{-1}\left(q\right)\tau_{a}.$$

where $M^{-1}\left(q\right)\tau_{d}$ is treated as the system disturbances with $M^{-1}\left(q\right)\tau_{a}$ the system input. Incorporating the FTO design skill in Reference [20], a basic FTO for robot disturbance is consequently designed in the following manner.

(15) $${\dot{z}}_{b1}=z_{b2}+K_{1}{\left\lfloor e_{b}\right\rceil }^{\alpha_{1}}$$

(16) $${\dot{z}}_{b2}=z_{b3}+M^{-1}\left(q\right)\tau_{a}+K_{2}{\left\lfloor e_{b}\right\rceil }^{\alpha_{2}}$$

(17) $${\dot{z}}_{b3}=K_{3}{\left\lfloor e_{b}\right\rceil }^{\alpha_{3}}$$

where $z_{b1}=\hat{q},z_{b2}=\hat{\dot{q}}$, $z_{b3}=M^{-1}\left(q\right){\hat{\tau }}_{d}$ and $e_{b}=q-\hat{q}$. $K_{1},K_{2},K_{3}\in R^{n\times n}$ are diagonal gain matrices. Moreover, the corresponding powers are selected as $\alpha_{1}=\alpha ,\alpha_{2}=2\alpha -1,\alpha_{3}=3\alpha -2$, and $\frac{2}{3}<\alpha <1$. The operator ${\left\lfloor \cdot \right\rceil }^{\alpha }$ is denoted as

(18) $${\left\lfloor x\right\rceil }^{\alpha }={\left|x\right|}^{\alpha }\mathrm{sgn}\left(x\right),x\in R^{n}\;\mathrm{and}\;\alpha >0.$$

Consequently, the disturbance observation ${\hat{\tau }}_{d}$ is computed as

(19) $${\hat{\tau }}_{d}=M\left(q\right)z_{b3}.$$

The proposed third-order FTO can simultaneously estimate the joint velocity $\dot{q}$ and disturbance torque ${\hat{\tau }}_{d}$. Indeed, the joint velocity $\dot{q}$ could be immediately acquired from the robotic control system, implying the basic FTO defined in Equations (15)–(17) could be tailored with both the observer order and computation burden reduction. Thus, a reduced-order FTO could be developed as

(20) $${\dot{z}}_{r1}=z_{r2}+M^{-1}\left(q\right)\tau_{a}+K_{1}{\left\lfloor e_{r}\right\rceil }^{\alpha_{1}}$$

(21) $${\dot{z}}_{r2}=K_{2}{\left\lfloor e_{r}\right\rceil }^{\alpha_{2}}$$

where $z_{b1}=\hat{\dot{q}},z_{b2}=M^{-1}\left(q\right){\hat{\tau }}_{d}$ and $e_{b}=\dot{q}-\hat{\dot{q}}$. $\alpha_{1}=\alpha ,\alpha_{2}=2\alpha -1$, $\frac{1}{2}<\alpha <1$. The calculation of the disturbance estimation resembles that given in Equation (19), i.e.,

(22) $${\hat{\tau }}_{d}=M\left(q\right)z_{r2}.$$

The reduced-order observer should have a quicker response than the previous design, which benefits the disturbance observation. However, it is worth noting that the inversion of the inertia matrix M(q) is involved in the observer design, which is quiet a troublesome issue for algorithmic computation. Specifically, the matrix inversion has a cubic complexity, which inevitably brings heavy computational burden in the case of robots with large DOFs. Generally speaking, the observed system defined in Equation (14) containing the inversion of M(q) should be responsible for the matrix inversion in the designed observer. In order to circumvent the matrix inversion, it is necessary to rearrange the original system from Equation (14) into a transformed equation with different state variables. Multiplying both sides of Equation (14) with M(q) yields

(23) $$M\left(q\right)\ddot{q}=\tau_{a}+\tau_{d}.$$

Additionally, the left side of Equation (23) could be altered using the generalized momentum *p*, namely

(24) $$\dot{p}-\dot{M}\left(q\right)\dot{q}=\tau_{a}+\tau_{d}.$$

**Figure 1:**
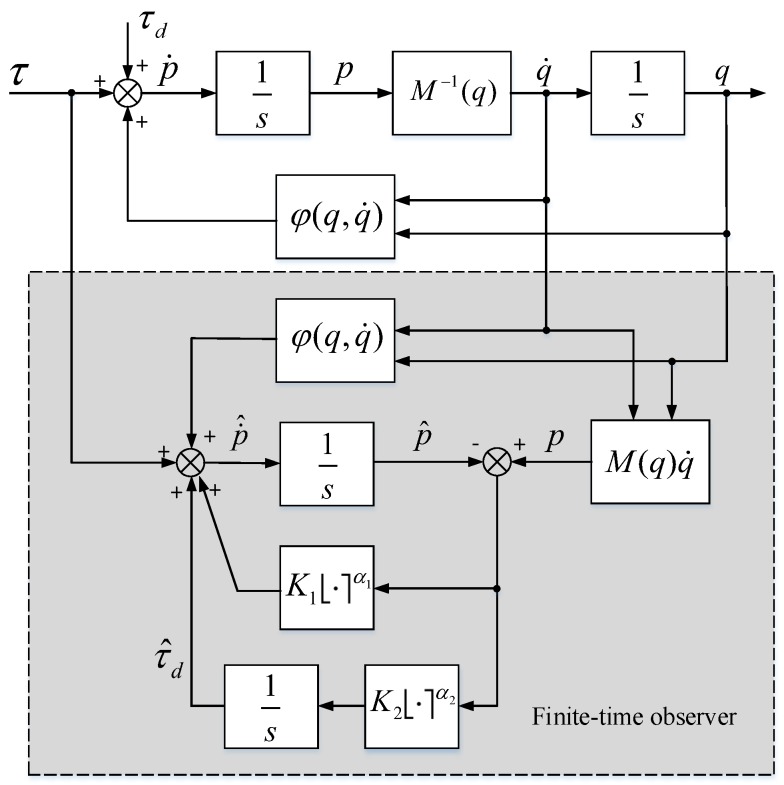
A block diagram of the proposed robotic disturbance finite-time observer (FTO).

Reorganizing Equation (24) and employing Property 1, the derivative of the generalized momentum *p* is rewritten as

(25) $$\dot{p}=\tau_{d}+\tau_{p}$$

where $\tau_{p}=\dot{M}\left(q\right)\left(q\right)+\tau_{a}=\tau +\varphi \left(q,\dot{q}\right),\varphi \left(q,\dot{q}\right)=C^{T}\left(q,\dot{q}\right)\dot{q}-G\left(q\right)-\tau_{f}$. The system to observe is now revised and has different state variables. Accordingly, a modified second-order FTO could be defined as follows.

(26) $${\dot{z}}_{m1}=z_{m2}+\tau_{p}+K_{1}{\left\lfloor e_{m}\right\rceil }^{\alpha_{1}}$$

(27) $${\dot{z}}_{m2}=K_{2}{\left\lfloor e_{m}\right\rceil }^{\alpha_{2}}$$

where $z_{m1}=\hat{p},z_{m2}={\hat{\tau }}_{d}$ and $e_{m}=p-\hat{p}$. The block diagram of the proposed FTO to estimate robotic disturbance is depicted in Figure 1.

To this end, three different types of FTO for robot disturbance estimation are introduced with a progressive optimization. The obtained FTO given in Equations (26) and (27) is structurally similar to the GMO defined in Equations (5) and (6) as both observer shares the same system states and input. Particularly, if we set α=1, then the designed FTO restores to an ESO formulation defined in Reference [10]. In other words, the ESO expression given in Reference [10] is a special case of the proposed FTO. As declared in Reference [10], ESO outperforms the popular GMO in disturbance observation in robot manipulator settings. It is reasonable to believe that the performance of FTO proposed in our paper should exceed that of both ESO and GMO.

### 3.2. Stability and Convergence of FTO

As the proposed observer is rooted in a general FTO design scheme, the stability and convergence of the presented DOB are evidently guaranteed. For completeness and readability, the error dynamics of FTO is inspected with a convergence analysis. The observation errors are given as

(28) $${\dot{e}}_{p}=e_{d}-K_{1}{\left\lfloor e_{p}\right\rceil }^{\alpha_{1}}$$

(29) $${\dot{e}}_{d}=-K_{2}{\left\lfloor e_{p}\right\rceil }^{\alpha_{2}}$$

where $e_{p}=p-\hat{p},e_{d}=\tau_{d}-{\hat{\tau }}_{d},e=\left[e_{p}\;e_{d}\right]$.

We will first prove the stability of the proposed observer. Consider the following Lyapunov function candidate:

(30) $$\begin{array}{cc} V & =\sum_{i=1}^{n}k_{2i}\int_{0}^{e_{pi}}{\left\lfloor \tau \right\rceil }^{\alpha_{2}}d\tau +\frac{e_{d}^{T}e_{d}}{2}\\ & =\sum_{i=1}^{n}\frac{k_{2i}}{\alpha_{2}+1}e_{pi}^{\alpha_{2}+1}+\frac{e_{d}^{T}e_{d}}{2} \end{array}$$

where $k_{2i}$ is the *i*th diagonal element of $K_{2}$. Then its derivative is given as

(31) $$\begin{array}{cc} \dot{V} & =\frac{\partial V}{\partial e^{T}}\dot{e}\\ & =\sum_{i=1}^{n}k_{2i}{\left\lfloor e_{pi}\right\rceil }^{\alpha_{2}}\left(e_{di}-K_{1}{\left\lfloor e_{pi}\right\rceil }^{\alpha_{1}}\right)-e_{d}^{T}K_{2}{\left\lfloor e_{p}\right\rceil }^{\alpha_{2}}\\ & =e_{d}^{T}K_{2}{\left\lfloor e_{p}\right\rceil }^{\alpha_{2}}-\sum_{i=1}^{n}k_{1i}k_{2i}{\left|e_{pi}\right|}^{\alpha_{1}+\alpha_{2}}-e_{d}^{T}K_{2}{\left\lfloor e_{p}\right\rceil }^{\alpha_{2}}\\ & =-\sum_{i=1}^{n}k_{1i}k_{2i}{\left|e_{pi}\right|}^{\alpha_{1}+\alpha_{2}}\leq 0. \end{array}$$

where $k_{1i}$ is the *i*th diagonal element of $K_{1}$. The only invariant set is the origin $e_{p}=e_{d}=0$. According to LaShalle theorem, the asymptotic convergence of *e* to zero is guaranteed.

Next, we will prove the finite-time convergence of the observer. According to Definition 2, Equations (28) and (29) are homogeneous of degree α−1 with respect to the weights $\left\{r_{1},r_{2}\right\}=\left\{1,\alpha \right\}$. Considering α<1, thus, Equations (28) and (29) have a negative homogeneity. From Theorem 1, it follows that the error system is globally FTS. In other words, the estimation errors will vanish within a finite time. To this end, it could be concluded that the proposed FTO is stable and with finite-time convergence performance.

## 4. Simulation Results

**Figure 2:**
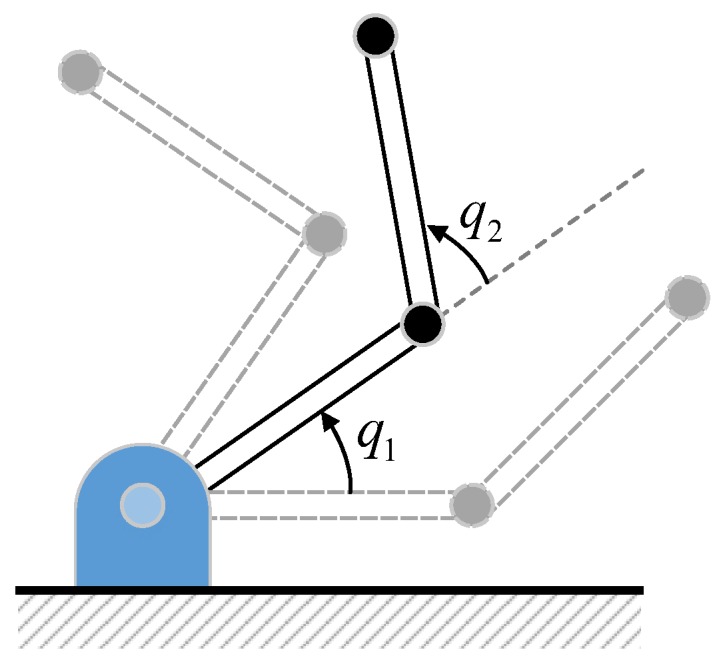
The studied two-link manipulator for simulation. The initial and end configurations for robot movement are in dashed lines.

In order to demonstrate the effectiveness and advantage of the proposed FTO algorithm in estimating robotic disturbance, a simulation was conducted on a simple 2-DOF planar manipulator vertical to the ground as depicted in Figure 2.

The studied manipulator is assumed to have a simple mass distribution, i.e., the mass of each link is concentrated as a point at the rod end. For simplicity, the friction effect will not be considered. The corresponding matrices in Equation (1) for this 2-DOF robot are expressed as

(32) $$M\left(q\right)=\left[\begin{array}{cc} \theta_{1}+2\theta_{2}\cos\left(q_{2}\right)\; & \theta_{3}+\theta_{2}\cos\left(q_{2}\right)\\ \theta_{3}+\theta_{2}\cos\left(q_{2}\right)\; & \theta_{3} \end{array}\right]$$

(33) $$C\left(q,\dot{q}\right)=\theta_{2}\sin\left(q_{2}\right)\left[\begin{array}{cc} -2\dot{q_{2}}\; & -\dot{q_{2}}\\ {\dot{q}}_{1}\; & 0 \end{array}\right]$$

(34) $$G\left(q\right)=\left[\begin{array}{c} \theta_{4}\cos\left(q_{1}\right)+\theta_{5}\cos\left(q_{1}+q_{2}\right)\\ \theta_{5}\cos\left(q_{1}+q_{2}\right) \end{array}\right]$$

where

(35) $$\left\{\begin{array}{c} \theta_{1}=l_{2}^{2}m_{2}+l_{1}^{2}\left(m_{1}+m_{2}\right)\\ \theta_{2}=l_{1}l_{2}m_{2}\\ \theta_{3}=l_{2}^{2}m_{2}\\ \theta_{4}=\left(m_{1}+m_{2}\right)l_{1}g_{0}\\ \theta_{5}=m_{2}l_{2}g_{0} \end{array}\right.$$

with $l_{i},m_{i}\;\left(i=1,2\right)$ as the length and mass for the *i*th link, respectively, and $g_{0}$ the gravitational acceleration. The manipulator performs point-to-point motion with two predefined configurations $q_{s}={\left[0,\;\pi /4\right]}^{T}$ and $q_{e}={\left[\pi /3,\;\pi /2\right]}^{T}$. For a smooth tracking trajectory generation, fifth polynomial interpolation is used to ensure a continuous reference acceleration. Without the loss of generality, the implemented controller for this example is merely a proportional–integral–derivative (PID) controller. Moreover, additional noises with a normal distribution are artificially imposed on the measurement signals. Specifically, $n_{q}∼N\left(0,7.56^{-10}\right)$ is the noise for joint position measurement with $n_{\tau }∼N\left(0,0.01\right)$ for torque. The simulation was based on MATLAB using a PC with a 3.5 GHz processor and 4 G memory.

As human–robot interaction is increasingly demanded in both domestic and industrial application, the necessity to quickly and accurately acquire the estimation of an interaction force is strengthened. Delayed or mismatched estimations may hinder the robot to sense its environment and human force intention. In this example, two prototypes of impact forces are employed with one step signal and the other ramp signal. The step signal may represent the fierce collision force, and the slop signals can be due to the normal interaction force. The step external torques are imposed on the time interval [1,1.8] seconds, and the slop impact forces are exerted during the time interval [0.8,1.6] seconds.

The proposed FTO method is then employed to estimate the external impact force and compared with other existing observers including NDOB [5], GMO [9], and ESO [10]. The gain matrix is selected to be diag{4,4} for NDOB and diag{50,50} for GMO. For ESO, its two gain matrices are chosen to be diag{200,200} and diag{10,000, 10,000}. When it comes to FTO coefficients selection, it contains more coefficients to be tuned. For simplicity, FTO shares the same gain matrices with ESO, namely $K_{1}=\mathrm{diag}\left\{200,200\right\}$ and $K_{2}=\mathrm{diag}${10,000, 10,000}. The power coefficient is chosen to be α= 0.9. As noted in a previous section, when α=1, FTO formulation degrades to ESO. However, this slight change in system powers significantly improves the performance of the estimation accuracy and convergent rate. Notice that the related parameters of these observers are tuned such that a similar noise level in residual signals is achieved.

**Figure 3:**
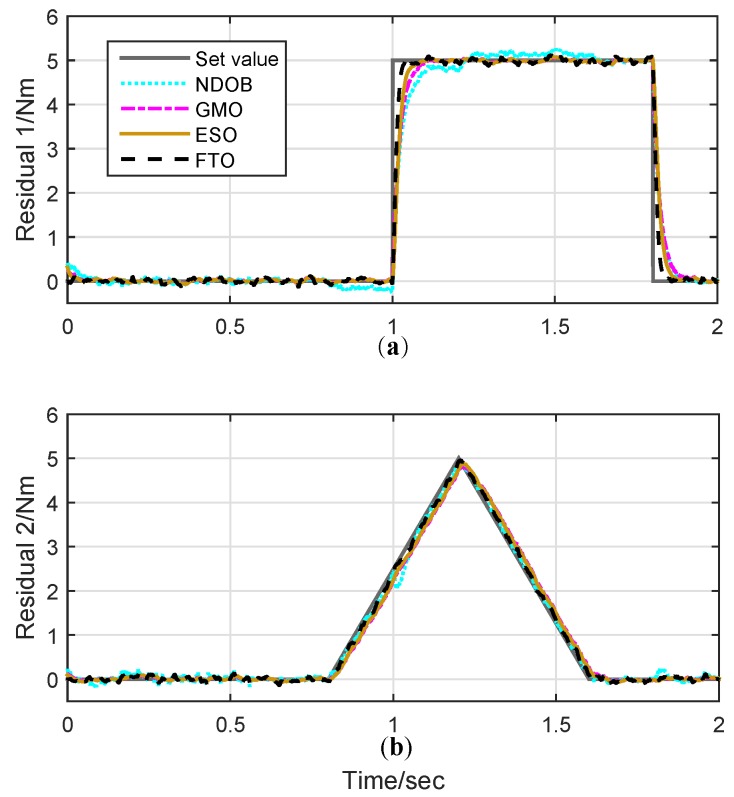
Estimation of external torque.(**a**) External disturbance observation results of step response signal for joint 1; (**b**) external disturbance observation results of slop signal for joint 2.

**Figure 4:**
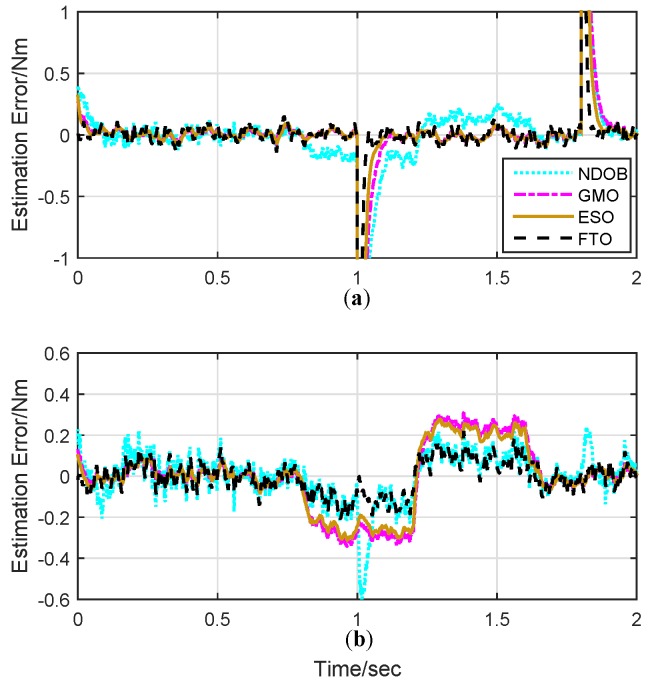
The disturbance estimation errors of external torque. (**a**) External disturbance estimation errors for joint 1; (**b**) external disturbance estimation errors for joint 2.

The time profiles of the observation results are shown in Figure 3, and the corresponding estimation error is also plotted in Figure 4. As it can be seen from Figure 3, FTO provides the quickest response compared to other DOBs thanks to the adoption of finite-time design, while ESO has better convergent rate compared to GMO and NDOB. Moreover, FTO also shows smaller estimation errors, especially for the dynamic force estimation. As shown in the bottom view of Figure 4, the FTO estimation error is comparatively smaller than that of both GMO and ESO in simulation. This fact demonstrates the fast convergence of the proposed FTO in disturbance observing. NDOB seems to share the same performance in slop disturbance tracking with FTO. However, NDOB is prone to sharp changes in acceleration as joint 2 undergoes violent acceleration processes near 1 and 1.8 seconds induced by the step disturbance on joint 1.

Table 1 details the specific estimation performance of these DOBs with different evaluation indices. The estimation errors for joint 1 and 2 are listed in the second and third column, respectively. It is clear that the proposed FTO outperforms other methods in minimizing the estimation error as FTO can track the real value with fast response. It should be pointed out that the model errors will be included in the lumped disturbances estimation in all of these DOBs. In order to realize an accurate estimation of the external impact, the model error should be kept under a low level. The last column in Table 1 illustrates the execution time per iteration of each observer. As the matrix inversion is required in NDOB, it takes the most computational time. Compared with GMO and ESO, FTO method costs slightly more computational time as a result of the power operation.

## 5. Conclusions

In this paper, a finite-time observer is designed to estimate the unknown disturbances imposed on a robotic manipulator. By reducing the observer order and reselecting the state variables, three types of FTO were iteratively designed and gradually optimized in order to ease the computation burden.

The adoption of the proposed FTO provides the disturbance estimation with a fast and accurate tracking capability, along with the avoidance of the joint acceleration calculation and matrix inversion. Compared with the existing DOBs of which the convergence rate is at best exponential, FTO is able to force the estimation error to converge to zero within a finite time. Although FTO has a more complex formulation and thus a slightly increased computational time cost, the simulation results demonstrate that FTO has the quickest response to disturbance and the lowest estimation error. Future work is to apply the proposed observer to the real manipulator with applications in collision detection and disturbance observer-based control. Moreover, theoretical work to adaptively handle robot model uncertainties will also be considered.

## Figures and Tables

**Table 1:** A comparison of nonlinear disturbance observer (NDOB), generalized momentum observer (GMO), external state observer (ESO), and FTO methods.

Method	RMSE1 (Nm)	RMSE2 (Nm)	$T_{e}$ (μs)
NDOB	0.5361	0.1204	13.50
GMO	0.5086	0.1634	5.79
ESO	0.4802	0.1495	7.72
FTO	0.4072	0.0805	9.64
